# Characterizing Active Statewide Surgical Quality Collaboratives Across the United States

**DOI:** 10.1097/AS9.0000000000000707

**Published:** 2026-09-04

**Authors:** Lisha Chen, Alison S. Baskin, Lesly A. Dossett, Chandler S. Cortina

**Affiliations:** * Division of Surgical Oncology, Department of Surgery, Medical College of Wisconsin, Milwaukee, WI; † Department of Surgery, University of California, San Francisco, CA; ‡ Center for Healthcare Outcomes and Policy, University of Michigan, Ann Arbor, MI; § Department of Surgery, University of Michigan, Ann Arbor, MI; ‖ Medical College of Wisconsin Cancer Center, Milwaukee, WI.

## INTRODUCTION

Surgical quality collaboratives (SQCs) are multi-institutional partnerships designed to improve patient outcomes through data sharing and joint learning.^1^ SQCs have improved surgical outcomes, strengthened safety culture, and enhanced multidisciplinary communication.^2–4^ However, the national landscape of SQCs in the United States has not been fully characterized, limiting policymakers’, healthcare systems’, and surgical leaders’ ability to evaluate infrastructure needs and inform strategies to expand successful SQC models. Therefore, we reviewed active statewide SQCs in the United States to define their characteristics and describe quality improvement (QI) initiatives.

## METHODS

We conducted a national descriptive study of US SQCs (December 2025–January 2026) using information from publicly available SQC websites, supplemented by a survey administered to SQC leadership to clarify and supplement online data (Supplemental Text 1, https://links.lww.com/AOSO/A669). Two reviewers independently extracted data from SQC websites. For inclusion, SQCs had to be multidisciplinary, statewide, and have a primary focus of improving surgical outcomes. SQCs were classified as ‘active’ if they had ongoing QI activities or meetings (confirmed by website and/or survey response). Variables extracted included operational status, number of participating hospitals, funding, leadership structure, data abstraction practices, and QI initiatives. Characteristics of active collaboratives were summarized along with recently completed and ongoing QI initiatives. This study was deemed exempt by the Medical College of Wisconsin Institutional Review Board.

## RESULTS

**FIGURE 1.**
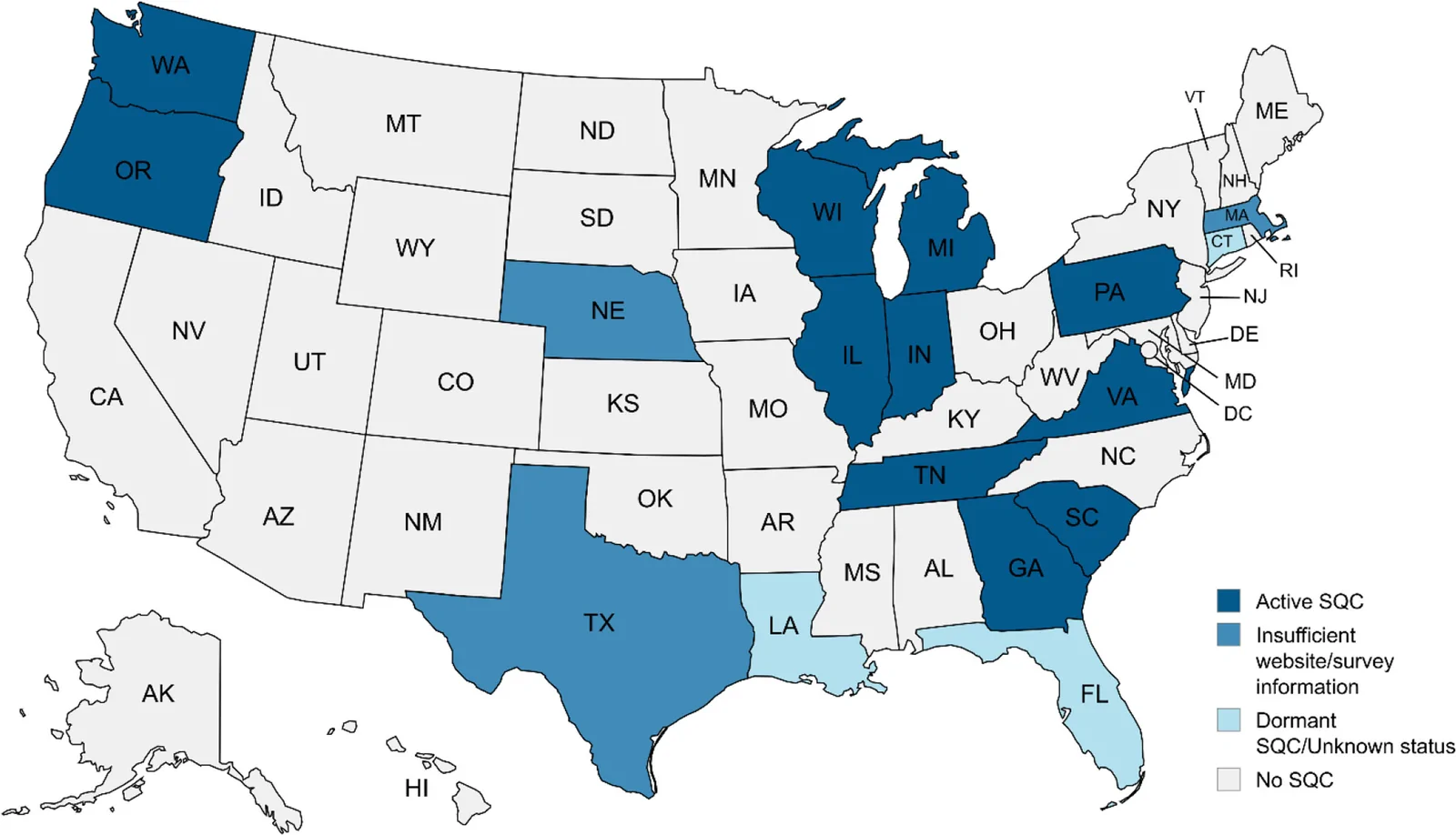
Statewide surgical quality collaboratives (SQCs) within the United States as of January 2026.

Seventeen states (n = 17/50, 34%) had an online SQC reference and received a survey (14/17 (82%) responded). Six SQCs were excluded due to insufficient website and survey information (n = 3) or unknown/dormant operational status (n = 3). One spanned 2 states (Illinois and Indiana). There were 10 active SQCs, established between 2003 and 2017 (Fig. 1).

Most SQCs (40%) included 11–20 hospitals (comprising approximately 25% of statewide hospitals), while larger SQCs included >50 participating hospitals (comprising 75%–100% of hospitals). Regarding leadership structure, 90% involved physicians and 30% involved nurses and/or advanced practice providers (some with dual leadership). Funding was from healthcare systems or participating hospitals (40%), independent insurance companies (40%), and state grants/contracts (30%), with 40% receiving funding from multiple sources. Half were affiliated with an academic institution. Surgical data were often collected by clinical reviewers (60%), but some relied on existing NSQIP infrastructure (30%) or nursing staff (10%). A minority (30%) used financial incentives to encourage hospital participation. Common initiatives focused on opioid reduction, surgical site infection prevention, venous thromboembolism reduction, and surgical oncology outcomes (Table 1).

**TABLE 1.** Select Examples of Surgical Quality Collaborative (SQC) Initiatives and Reported Outcomes in the United States From 2010 to 2025

Initiative Type	SQC Name(s)	Initiative Objective	Reported Outcome
Safety culture	Illinois Surgical Quality Improvement Collaborative	Created a statewide SQC incorporating a standardized data platform, quality improvement training and projects, and process improvement coaches and mentors.	Improved teamwork climate, safety climate, physician–nurse collaboration, and reporting of concerns.^2^
Health policy (Medicaid expansion)	Virginia Surgical Quality Collaborative (VSQC) and Tennessee Surgical Quality Collaborative (TSQC)	Compared readmissions, morbidity, and mortality before and after Medicaid expansion in a Medicaid expansion state (Virginia) and a non-expansion state (Tennessee).	VSQC observed fewer uninsured patients, lower 30-d readmission rates, and reduced morbidity following Medicaid expansion. No comparable improvements were observed in TSQC.^5^
Opioid stewardship	Michigan Surgical Quality Collaborative (MSQC)	Used patient-reported opioid consumption data to reduce postoperative opioid prescribing guidelines across participating hospitals, followed by reassessment of opioid use patterns.	Patient-reported opioid use decreased for 9 of 16 procedures, allowing further reductions in prescribing recommendations.^6^
Surgical quality improvement	Michigan Surgical Quality Collaborative (MSQC)	Conducted quarterly collaborative meetings to share best practices and develop quality improvement strategies aimed at reducing surgical complications.	Compared with non-Michigan American College of Surgeons NSQIP hospitals, MSQC hospitals demonstrated lower morbidity and complication rates, with no difference in mortality.^4^
Breast Surgery	Surgical Collaborative of Wisconsin (SCW)	Introduced benchmark performance reports and collaborative meetings to share quality improvement measures and guideline updates across participating hospitals.	Re-excision rates decreased significantly among SCW hospitals, while rates remained unchanged at non-SCW hospitals.^7^
Breast Surgery	Surgical Collaborative of Wisconsin (SCW)	Evaluated rates of sentinel lymph node biopsy (SLNB) during breast-conserving surgery for DCIS between 2017 and 2023.	SLNB rates decreased overall, although substantial interhospital variation remained. Findings prompted a statewide initiative focused on surgeon education and collaborative efforts to standardize practice.^8^
Colorectal Surgery	Virginia Surgical Quality Collaborative (VSQC)	Implemented an ERAS protocol across participating hospitals.	Following implementation, laparoscopic colectomy patients experienced shorter length of stay, fewer 30-day readmissions, and fewer complications. Open colectomy patients also demonstrated reduced length of stay.^3^

DCIS indicates ductal carcinoma in situ; ERAS, enhanced recovery after surgery; SLNB, sentinel lymph node biopsy.

## DISCUSSION

In this national study of statewide SQCs, we found only 10 US states have an active SQC. Although SQCs varied in several characteristics, some elements were consistently observed, including physician leadership and funding from healthcare systems and insurers.

Inactive/dormant SQCs suggest challenges in long-term sustainability. Potential difficulties may include funding challenges, as several active SQCs reported having no consistent/dedicated funding source. Some SQCs described potential expansion to nearby states to create regional collaboratives, indicating participation challenges may limit viability. Leveraging existing data platforms, partnering with larger academic institutions, or developing regional collaboratives represent potential strategies to improve sustainability.

Several active SQCs reported improved clinical outcomes after targeted initiatives. Pennsylvania and Michigan SQCs have demonstrated decreased opioid use following opioid reduction initiatives.^6^ In Wisconsin, there was a decrease in breast lumpectomy margin re-excision rates among hospitals that participated in QI meetings and performance benchmarking activities.^7,8^ SQCs may also facilitate clinical research as demonstrated in Virginia, where the SQC leveraged its data infrastructure to identify decreases in perioperative morbidity and readmission rates following Medicaid expansion.^5^ Ultimately, SQCs facilitate collaboration among surgical teams and hospitals through learning sessions, plan-do-study-act cycles, and multidisciplinary QI teams, which promote shared learning, dissemination of best practices, and a collegial culture among participants.^9^

Our study is limited by its reliance on publicly available information and survey responses, which are subject to reporting bias. The availability of updated websites and survey responsiveness likely reflects operational status. Additionally, we did not directly assess barriers or facilitators of success or evaluate dormant SQCs, but these are the focus of future work. Despite these limitations, this study is the first to characterize active statewide SQCs in the United States.

Only 20% of US states have an active SQC, and long-term sustainability may be a challenge. Future work to understand factors that can ensure long-term SQC success is essential to strengthen, expand, and amplify the clinical impact of SQCs.

## Supplementary Material



## References

[1] Wandling MW Minami CA Johnson JK. Development of a conceptual model for surgical quality improvement collaboratives: facilitating the implementation and evaluation of collaborative quality improvement. JAMA Surg. 2016;151:1181–1183. 10.1001/jamasurg.2016.2817 PMC8944346 27604075

[2] Yuce TK Yang AD Johnson JK. Association between implementing comprehensive learning collaborative strategies in a statewide collaborative and changes in hospital safety culture. JAMA Surg. 2020;155:934–940. 10.1001/jamasurg.2020.2842 PMC7424544 32805054

[3] Hedrick TL Thiele RH Hassinger TE. Multicenter observational study examining the implementation of enhanced recovery within the virginia surgical quality collaborative in patients undergoing elective colectomy. J Am Coll Surg. 2019;229:374–382.e3. 10.1016/j.jamcollsurg.2019.04.033 31108195

[4] Campbell DA Jr Englesbe MJ Kubus JJ. Accelerating the pace of surgical quality improvement: the power of hospital collaboration. Arch Surg. 2010;145:985–991. 10.1001/archsurg.2010.220 20956768

[5] Turrentine FE Charles EJ Marsh KM. Impact of medicaid expansion on abdominal surgery morbidity, mortality, and hospital readmission. J Surg Res. 2023;291:586–595. 10.1016/j.jss.2023.06.047 PMC10529060 37540976

[6] Kulkarni AJ Gunaseelan V Brummett CM. Postoperative opioid consumption after discharge: an update from the michigan surgical quality collaborative registry. Ann Surg Open. 2024;5:e517. 10.1097/AS9.0000000000000517 PMC11661719 39711658

[7] Schumacher JR Lawson EH Kong AL. A statewide approach to reducing re-excision rates for women with breast-conserving surgery. Ann Surg. 2022;276:665–672. 10.1097/SLA.0000000000005590 PMC9529150 35837946

[8] Cortina CS Schumacher JR Cartmill RS. Sentinel node biopsy utilization in wisconsin women undergoing breast-conserving surgery for DCIS. Ann Surg Oncol. 2025;32:5446–5448. 10.1245/s10434-025-17590-5 PMC12210222 40450165

[9] Nadeem E Olin SS Hill LC. Understanding the components of quality improvement collaboratives: a systematic literature review. Milbank Q. 2013;91:354–394. 10.1111/milq.12016 PMC3696201 23758514

