# Supplementary material for: Characterizing Active Statewide Surgical Quality Collaboratives Across the United States
Source: Ann Surg Open. 2026 Sep 4;7(3):e707. doi: 10.1097/AS9.0000000000000707 (PMC13596941; doi:10.1097/AS9.0000000000000707)
Supplement: Supplementary file 1 [file as9-7-e707-s001.pdf]

Default Question Block

We are seeking to characterize the current landscape of statewide surgical quality collaboratives across the United States. Your help in providing information regarding the current status of these collaboratives is greatly appreciated. Please answer the following questions to the best of your knowledge and feel free to consult colleagues as needed.

This survey should take approximately 5-10 minutes. Participation in this survey is completely voluntary. All identifiable personal information will be removed. For questions or concerns, please contact: Alison Baskin at alison.baskin@ucsf.edu or Lisha Chen at lchen@mcw.edu.

Which state do you work in?

Does your state currently have an active statewide surgical quality collaborative?

- Yes (provide name)
- No
- Unsure

Did your state previously have a statewide surgical quality collaborative that is no longer active?

- ○ [ ] Yes (provide name)
- ○ No
- ○ Unsure

In what year was this collaborative established? (Leave blank if unsure)

[ ]

Approximately how many hospitals currently participate in this collaborative?

- ○ <10
- ○ 11-20
- ○ 21-50
- ○ >50
- ○ Unsure

Approximately what percentage of hospitals in the state currently participate in this collaborative?

- ○ 1-25%
- ○ 26-50%
- ○ 51-75%
- ○ 76-100%
- ○ Unsure

Who leads this collaborative? (Select all that apply)

- ☐ Physician (MD/DO)
- ☐ Nurse or Advanced Practice Provider (RN/NP/PA)
- ☐ Business Executive (not trained health care worker)
- ☐ Other (explain)
- ☐ Unsure

Who are the primary funder(s) of the collaborative? (Select all that apply)

- ☐ Insurer (e.g., Blue Cross Blue Shield)
- ☐ State grant or contract
- ☐ Private foundation or philanthropic organization
- ☐ Health system(s) / participating hospitals
- ☐ Other (explain)
- ☐ Unsure

Are there any other funders of this collaborative?

- ○ Yes (provide name)
- ○ No
- ○ Unsure

Is the collaborative affiliated with a specific academic

institution/university?

- ○ [ ] Yes (provide name)
- ○ No
- ○ Unsure

What are some of the collaborative's current and/or recent quality improvement initiatives (e.g. reducing surgical site infections, opioid reduction, colorectal outcomes, breast surgery practices)? (Leave blank if unknown)

[ ]

What is the mission statement of the collaborative, if available? (Leave blank if unknown)

[ ]

Who are the primary data abstractors for your collaborative?

- ○ Surgical clinical reviewers
- ○ Nursing staff
- ○ Other hospital-based staff (not nurses)
- ○ N/A - existing data infrastructure, no additional data collected
- ○ [ ] Other (explain)
- ○ Unsure

Do participating institutions receive a financial incentive for their involvement in the collaborative?

- ○ Yes, pay-for-participation
- ○ Yes, pay-for-performance
- ○ [ ] Yes, other (explain)
- ○ No
- ○ Unsure

Please share any additional information you may have regarding this surgical quality collaborative.

[ ]

Powered by Qualtrics
